# P69 COVID vaccine uptake in young persons on immunosuppression under the care of tertiary paediatric rheumatology service

**DOI:** 10.1093/rap/rkac067.069

**Published:** 2022-09-28

**Authors:** Rupanjana Choudhary, Rachel Smith, Elizabeth Smith, Samundeeswari Deepak, Kishore Warrier

**Affiliations:** University of Nottingham, Nottingham, United Kingdom; Nottingham University Hospitals, Nottingham, United Kingdom; Nottingham University Hospitals, Nottingham, United Kingdom; Nottingham University Hospitals, Nottingham, United Kingdom; Nottingham University Hospitals, Nottingham, United Kingdom

## Abstract

**Introduction/Background:**

COVID-19 disease, caused by SARS-CoV-2 virus, first emerged as a presentation of severe respiratory disease in 2019. Although children exhibit mild disease, concerns were raised about serious infection in immunocompromised children. The current national guidance recommends that immunocompromised children between 12 and 18 years of age should be offered three primary doses of the Pfizer BioNTech vaccine, 8 weeks apart, followed by a booster dose within 3 months. This audit looked at the awareness and uptake of this regime in young persons on immunosuppression. This information was disseminated to families during clinical contacts and automatic replies sent out from the team mailbox.

**Description/Method:**

To collect the data for the audit, we used an anonymous questionnaire to capture the following information:

• Diagnosis

• Immunomodulatory/Immunosuppressive treatment

• Their awareness of the regime for COVID vaccine (number of doses and interval)

• Number of vaccines taken

• If not taken, why?

The questionnaires were handed out to 31 patients attending Paediatric Rheumatology day-care and outpatient clinics over a two-week period for their response. There were no non-responders. Patients who were not on any immunosuppressant medication were excluded.

The split of their diagnoses and medications are summarised in tables 1 and 2:

Patients with JIA (55%) unsurprisingly dominated the cohort, followed by JSLE, JDM and scleroderma. Only 3/31 patients (10%) were on any form of systemic steroid therapy (all on oral Prednisolone less than 0.5 mg/kg). Majority of the patients (21/31 – 68%) were on Methotrexate, either on its own, or in combination with one of the biologic agents (one scleroderma patient was on MTX + MMF). 14/21 (45%) patients were on one of the biologic agents, of which only one patient was on Rituximab.

**Discussion/Results:**

The results showed that only 6/31 (19%) of patients/their carers knew the correct number (3 + 1) of COVID vaccines recommended in this cohort. 18/31 (58%) patients had received the vaccine, of which 4 patients refused further doses due to side effects they had experienced following the first dose. Of the 13 young people who did not take the vaccine, 9 patients (29%) were unsure whether they need the vaccine. They did not know the benefits of the vaccine and were afraid it either could have a negative effect on immunocompromised children or just were not aware of the guidelines. The other 4 (13%) participants did not want to take the vaccine due to their own personal beliefs.

Although concerns were raised about serious COVID-19 infection in immunocompromised children, there is currently very little evidence to suggest that the infection rate and severity of COVID-19 infection in children treated with Methotrexate or biologic agents is higher or the treatment needs to be discontinued or the doses adjusted. There is some emerging evidence to suggest that adults on Rituximab and to a lesser extend Mycophenolate may have increased risk of developing severe outcomes from COVID-19, which has not been replicated in children. The recommendation for third primary dose is based on preliminary results from UK studies of real-world vaccine effectiveness (VE) in persons who are immunosuppressed, suggesting a modest reduction in VE against symptomatic COVID-19. There is also emerging evidence that COVID vaccination significantly reduces the risk of Paediatric Inflammatory Multisystem Syndrome (PIMS-TS) in 12 – 18 year olds.

**Key learning points/Conclusion:**

Despite the notion that the team had disseminated information on the vaccine regime to patients, only one fifth of the patients/carers knew the exact regime for COVID vaccine recommended in this group of young people.

Just under 60% of the patients took the vaccine, with the rest choosing not to have it due to a mixture of lack of information and personal choice.

The action plan was drawn up to:

1. enquire about and document vaccination status, and provide advice and information during every clinical contact;

2. include COVID vaccine in education sessions by nursing team;

3. include the information as default in all clinic letters;

4. reaudit in six months.

**P69 Table 1:** Patients' diagnoses

Diagnosis	Number of respondents
Juvenile Idiopathic Arthritis (JIA)	17 (2 – uveitis) – 55%
Juvenile Systemic Lupus Erythematosus (JSLE)	5 – 16%
Juvenile Dermatomyositis (JDM)	3 – 10%
Localised scleroderma / Systemic Sclerosis	3 – 10%
ANCA Associated Vasculitis (AAV)	1 – 3 %
Systemic Autoinflammatory Disease	2 (both Mevalonate Kinase deficiency) – 6%

**P69 Table 2:** Patients' medications

Medications	Number of respondents
Systemic steroids (on top of other medicines)	3 (10%)
Methotrexate (MTX)	11 (35%)
Methotrexate + Adalimumab	6 (19%)
Mycophenolate Mofetil (MMF)	4 (13%)
MTX + MMF	1 (3%)
Adalimumab	3 (10%)
MTX + Infliximab	3 (10%)
Canakinumab	2 (6%)
Cyclophosphamide	1 (3%)

